# Country-led institutionalization of community health within primary health care: Reflections from a global partnership

**DOI:** 10.7189/jogh.11.03037

**Published:** 2021-03-01

**Authors:** Nicholas Leydon, Nazo Kureshy, Hannah-Sarah Dini, Rory Nefdt

**Affiliations:** 1 The Bill & Melinda Gates Foundation, Seattle, Washington, USA; 2 Social Solutions International, supporting USAID, Washington, D.C., USA; 3 Independent consultant, New York, New York, USA; 4 UNICEF, New York, New York, USA

Despite its foundational role for primary health care (PHC), community-level care has struggled for decades to be prioritized within PHC systems. At Astana in 2018 [1] and Alma Ata 40 years prior [2], national and global health leaders highlighted the need to make communities essential in PHC. Limited health spending is often directed to facility-based service delivery, such as hospitals, leaving the community health workforce, supply chains, and information systems underfunded and poorly integrated into PHC. In low income countries, approximately 65% of health spending is allocated to primary care with development assistance partners contributing up to 40% of PHC funding [3]. Development assistance between 2007-2017 targeting community health worker (CHW) projects accounted for 2.5% of the total development assistance for health [4]. Historically, funding for community health and its integration has resulted in vertical, patchwork services. Funding for community health is/remains primarily donor funded and channeled through vertical mechanisms, with varying levels of coordination and institutionalization, guided by national priorities and needs. More effective engagement of communities in both government and donor supported projects for PHC systems strengthening can help overcome these challenges.

The purpose of our multi-agency collaboration has been two-fold: support integration of community health into PHC systems in seven focal countries ^(^Bangladesh, Democratic Republic of the Congo, Haiti, Kenya, Liberia, Mali, and Uganda), and influence the global PHC agenda by elevating the importance of community health to accelerate Universal Health Coverage (UHC). The first objective of our collaboration is realized through catalytic partnerships among governments, trusted non-governmental partners, and communities to operationalize national policy and systems reform focusing on CHWs as an entry point [5]. The second objective of our collaboration supports the generation of new evidence and knowledge through grants to the Population Council [6] and Last Mile Health [7] to advance performance measurement, advocacy, and pathways to scale. Using our organizations’ convening processes as well as our relationships with governments and key partners, we proactively supported the growing country demand following the development and collective endorsement of Ten Principles to Institutionalize Community Health by 23 country delegations at the Institutionalizing Community Health Conference (Johannesburg, 2017) [8]. To achieve our goals, we have backed ongoing intra- and cross-country learning and created a virtual community of practice for over twenty countries [9].

**Figure:**
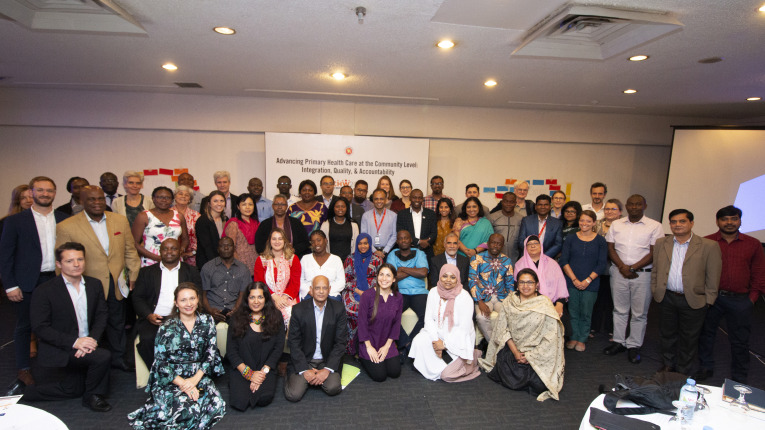
Photo: Instituting Community Health (ICH) partners and colleagues at the Community Health Worker Symposium in Dhaka, Bangladesh (November 2019); used with permission.

Over the past 40 years, countries have made some notable shifts in delivering PHC services at the community level in the pursuit of UHC. Initially led by trailblazers such as Bangladesh, other countries have realized the successful 10:1 return on investment for CHW programs [10], including Pakistan, Brazil, Rwanda, Ethiopia, and Liberia [11]. While some exemplar countries have managed to design and reinvent their CHW programs, many countries struggle with diverse challenges in program design, implementation, and financing [12]. Too frequently, the planning, budgeting, and implementation of community health programs are divorced from the PHC system. In addition, the promising practices of non-governmental partners are not integrated and scaled up as a part of national PHC priorities and implementation plans [13]. Fortunately, the trend is towards integration, which will rely on the following three areas of increased intellectual and financial investment.

## INVEST IN PUBLIC SECTOR COMMUNITY HEALTH PRIORITIES

First, countries that have been successful in strengthening PHC at the community level are those that have had government-led priority-setting backed by strong alignment within the MOH, with key non-governmental partners, and with donors. Ethiopia’s Health Extension Program, for example, is well-integrated into PHC and is now under further optimization to link the Women’s Development Army with the Health Extension Workers. Zambia has developed a new strategic plan, including a detailed financial projection of what it would cost to scale to 5000 Community Health Assistants. Finally, in Kenya, County Community Health Services Bills have been passed in subnational parliaments to provide legal recognition and regulation of community health services in support of a new community health strategy.

While these examples demonstrate country investments towards community health priorities, most countries do not yet have nationally coordinated and operationalized community health strategies. To close the gap, our partnership and other collaborators have developed platforms and tools to support country investments in community health. For example, the Community Health Roadmap [14] elevates national priorities for community health through donor collaboration, global advocacy, and cross-country learning. For countries seeking best practices, community health exemplars [15] and a set of implementation tools are available to accelerate progress. For example, Last Mile Health has leveraged more than a decade of learning in Liberia to develop a “reform cycle” that other countries can use for policy development. In addition, the Population Council has established a conceptual measurement framework to guide community health leaders eager to track data [16]. These documented exemplars and key tools, when used in support of national public health leadership, can establish a vision for all development partners.

## INVEST IN QUALITY

Second, as countries integrate and expand the package of services delivered by CHWs, a robust focus on quality at the community level must keep pace with equitable access [17]. Countries focused on achieving high quality services are simultaneously pursuing bundles of interventions, such as accessible workforce education platforms, supportive supervision, and quality improvement methods to amplify the effect of individual strategies [18]. Our partnership has learned from countries that have introduced quality improvement and accountability mechanisms for community health. The quality of Liberia’s Community Health Assistant program is monitored by combining routine data from its Community-Based Information System with a set of facility and community surveys to assess systems performance issues (eg, related to supervision, supply chain issues, and worker competency) [19]. In Kenya, sub-country and community-level quality improvement teams were established to implement continuous improvement methods, such as the use of community-generated data to monitor the performance and quality of community health services [20]. Given that community-based workers shoulder increasing service delivery responsibilities, on-demand virtual education platforms, such as the Community Health Academy and innovative coaching tools, will be critical to achieve optimal service quality at scale.

## INVEST IN GOVERNANCE

Lastly, building accountability mechanisms that drive locally acceptable solutions is critical for success. The community agents must have decision space to affect change. Uganda’s National Community Health Learning and Improvement Initiative (NACHLII), for example, strengthens community health leadership, governance, and multi-sectoral collaboration throughout the entire health system (national to community level) by investing in the active engagement of communities to increase participation, ownership, and their capacity to be agents of their own health [21]. UNICEF’s Child Friendly Community Real Time Monitoring program in Democratic Republic of the Congo (DRC), Togo, Guinea, Chad, and Liberia annually assesses the functionality of community governance mechanisms [22]. In addition, DRC has installed 47 000 community outreach units (Cellule d’Animation Communautaire) – 30% of which are currently considered functional according to national policy – comprised of stakeholders from different sectors to set ambitious targets linked to national standards [23]. These units have provided critical infrastructure and governance during DRC’s Ebola and COVID-19 responses.

To engage stakeholders and drive accountability, community participation must go beyond traditional demand generation efforts. Creating durable governance mechanisms that routinely solicit and respond to voices from community members, especially patients, will help ensure that efforts to institutionalize community and PHC services are acceptable and accountable to the communities. For example, Ethiopia is using a community scorecard for citizen feedback to Primary Health Care Unit governance systems to promote accountability and improved implementation [24]. We acknowledge that most health systems do not collect rapid pulse community feedback; however, we nevertheless advocate for this capability because it would enable more responsive and efficient service delivery.

Fortunately, these investments in national priority-setting, service quality, and governance will pay significant dividends in the years to come. Countries can leverage global mechanisms like the Global Financing Facility, GAVI, and the Global Fund to accelerate their PHC agendas, particularly at the community level. And global experts, such as the Quality of Care Network, are well poised to propose quality of care standards for community-level services, similar to facility-based care. Guidelines from the World Health Organization (WHO) provided [25] the evidence for widespread support for the first ever resolution on community health workers passed at the 72nd World Health Assembly [26]. Countless tools to support the costing, policy path, and measurement of CHW programs now exist and require that countries and development partners judiciously apply those tools rather than overwhelming short-staffed ministries. With increased pressure from Ebola and COVID-19, virtual learning platforms and distance supervision have become more sophisticated to address rapidly evolving scenarios. Global partnerships, such as ours, have enabled south-to-south dialogue, accelerating the rapid diffusion of successful ideas. Finally, we recognize that a growing movement of countries, donors, and development partners must rally around national priorities for PHC, striking a balance between cross-cutting and vertical investments from donor and domestic resources, national and local partnerships in a whole of society approach for health systems, and integration of CHWs and broader community engagement in PHC systems [27]. Our collaboration will continue to learn from and support countries as they expand national PHC priorities with communities at the center.
